# Clinical Study of Laser Treatment for Frenectomy of Pediatric Patients

**DOI:** 10.5005/jp-journals-10005-1449

**Published:** 2017-02-27

**Authors:** Sayaka Komori, Kousuke Matsumoto, Kenji Matsuo, Hiroaki Suzuki, Takahide Komori

**Affiliations:** 1 Attending Staff, Department of Oral and Maxillofacial Surgery, Graduate School of Medicine, Kobe University, Chuo-ku, Kobe, Japan; 2 Assistant Professor, Department of Oral and Maxillofacial Surgery, Graduate School of Medicine, Kobe University, Chuo-ku, Kobe, Japan; 3 Undergraduate Student, Department of Oral and Maxillofacial Surgery, Graduate School of Medicine, Kobe University, Chuo-ku, Kobe, Japan; 4 Lecturer, Department of Oral and Maxillofacial Surgery, Graduate School of Medicine, Kobe University, Chuo-ku, Kobe, Japan; 5 Professor, Department of Oral and Maxillofacial Surgery, Graduate School of Medicine, Kobe University, Chuo-ku, Kobe, Japan

**Keywords:** CO_2_ laser, Lingual frenectomy, Maxillary labial frenectomy.

## Abstract

**Aim:**

To suggest regarding the timing of oral surgery and laser treatment for frenulum abnormalities in the pediatric population.

**Materials and methods:**

We investigated the sex, age, frenulum site, reason for consultation, treatment method, and prognosis of 35 patients aged 15 years or younger and who were examined at our hospital for the chief complaint of frenulum abnormality.

**Results:**

A total of 21 (mean age, 6.0 years) of the 35 patients underwent frenectomy using a carbon dioxide (CO_2_) laser. Of these, 7 patients (mean age, 2.8 years) underwent the procedure with general anesthesia and 14 patients (mean age, 7.6 years) underwent the procedure with local anesthesia. The surgical site was the lingual frenulum in 15 patients and the maxillary labial frenulum in 6 patients. No adverse events were intraoperatively reported in any of the patients, and the procedure was quickly and safely performed. The mean postoperative follow-up period was 4.6 months, and readhesion was noted in one patient (4.8%). The most common reason cited for not undergoing frenectomy in the 14 patients (mean age, 3.4 years) was the young age of the child.

**Conclusion:**

Retrospective study of pediatric patients with frenulum abnormalities demonstrated the usefulness of the CO_2_ laser in performing frenectomy and offered suggestions regarding the timing of this procedure.

**Clinical significance:**

Frenectomy performed using a CO_2 _laser for pediatric patients is a useful, simple, and safe treatment method.

**How to cite this article:**

Komori S, Matsumoto K, Matsuo K, Suzuki H, Komori T. Clinical Study of Laser Treatment for Frenectomy of Pediatric Patients. Int J Clin Pediatr Dent 2017;10(3):272-277.

## INTRODUCTION

Most frenulum abnormalities occur in the lingual or maxillary labial frenulum. In the lingual frenulum, adhesion to the proglossis impairs tongue movement, causing problems, such as suckling, articulation, and speech disorders. In the maxillary labial frenulum, high adhesion to the alveolar portion causes diastema of the dentition and eruption site abnormalities in the central incisors.^1^ Although treatment might be required if these symptoms are observed, this is often difficult due to problems in gaining the understanding and cooperation of the patient because these are mostly infants or early school year-aged children. In addition, the pediatric patients could even be subjected to psychological trauma by the distress experience of the stressful treatment from dentist; hence, a simple method of treatment that offers reliable effects is needed.

As the CO_2_ laser has a wavelength of 10.6 μm, it shows excellent hemostatic ability with minimal heat damage to the surrounding tissues, and it is used for the resection and vaporization of oral soft tissue because it can do so without coming into contact with the affected site.^2,3^ At our hospital, the CO_2_ laser is used for many procedures, including soft tissue tumor resection, mucositis vaporization, and mucous cyst extirpation, as well as commonly for frenectomy.^4,5^ Frenulum abnormalities are generally difficult to judge and treat in pediatric patients. This clinical retrospective study investigated pediatric patients with frenulum abnormalities in our hospital and demonstrated the disease state and the usefulness of CO_2_ laser treatment.

## MATERIALS AND METHODS

**Figs 1A to D:**
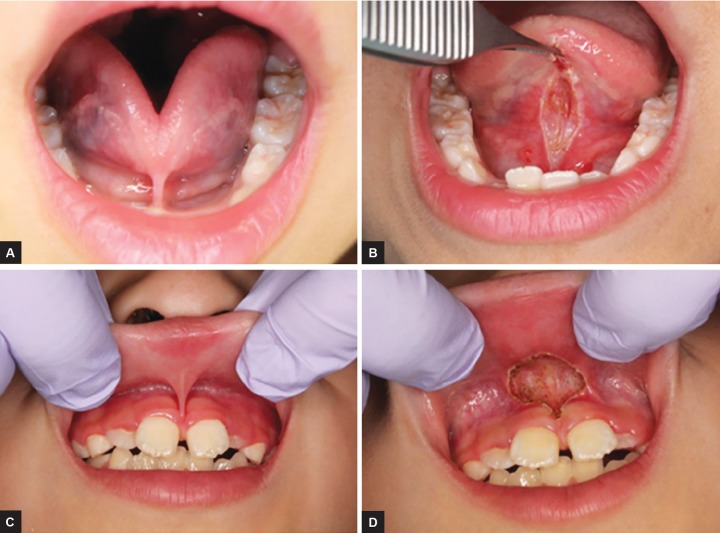
Example of procedures. (A) A preoperative view of a lingual frenulum attachment; (B) immediately after a lingual frenectomy with the CO_2_ laser, the tongue is able to move freely and the range of movement is greatly increased; (C) a preoperative view of a maxillary frenulum attachment; and (D) the final rhomboidal laser cut does not require sutures or periodontal dressing. Postoperative hemostasis is optimum

We assessed the sex, age, frenulum site, reason for consultation, classification, prognosis, and complications of 35 patients aged 15 years or younger who were examined at our hospital for undergoing frenectomy in the 5-year period from March 2010 to March 2015. Ito’s classifications^6^ and Rui’s classifications^7^ were used as references of the classifications for lingual and maxillary labial frenulum respectively (Table 1). The procedure involved infiltrating about 1 mL of anesthesia into the frenulum before applying a continuous wave of the CO_2_ laser at 2 to 5 W for approximately 60 seconds. For the lingual frenulum, a suture thread was occasionally placed around the proglossis to apply traction and sufficiently extend it before applying the laser to its base (Figs 1A and B). For the maxillary labial frenulum, the upper lip was retracted with the fingers, and the frenulum was extended while the laser was applied to the gingival aspect of the alveolar part to which traction was being applied (Figs 1C and D). After resection, suture was required in seven of the 15 patients with lingual frenectomy and one of the 6 patients with maxillary labial frenectomy. Postoperatively, prophylactic antibiotics and analgesics were administered, and all patients were given instructions on extension exercises for the tongue or upper lip to prevent re-adhesion. This study was a retrospective study. Informed consent was obtained from all the patients and their parents after giving them a sufficient explanation.

**Table 1:** **Table 1:** Representative classifications of frenulum

*Types*		*Classifications*	
		*Ito’s lingual frenulum*	
I		Despite sufficiently opening the mouth and elevating the lingual apex, the lingual apex does not reach the palate. The lingual apex is constricted and appears to be two apexes	
II		Despite elevating the lingual apex, it only lifts slightly higher than the occlusal plane	
III		The lingual apex can hardly be elevated at all	
		*Rui’s maxillary labial frenulum*	
I		Alveolar musoca	
II		Gingival insertion	
III		Interdental papilla	
IV		Transpapillar	

## RESULTS

The study sample comprised 20 boys and 15 girls aged between 1 month and 14 years. The frenulum site was the lingual frenulum in 27 patients and the maxillary labial frenulum in 8 patients. The patients’ reasons for consultation were as follows: being advised to get a regular checkup at a dental clinic or school (13/35 patients; 37.1%), speech disorders (7 patients; 20.0%), cosmetic problems needing orthodontic treatment (5 patients; 14.3%), being advised by parents or people around them (4 patients; 11.4%), eating/suckling disorder (3 patients; 8.6%), postresection recurrence (1 patient; 2.9%), and other reasons [(2 patients; 5.7%); (Table 2)].

**Table 2:** **Table 2:** Patients reasons for consultation

		*Lingual frenulum*		*Maxillary frenulum*		*Total*	
Regular checkup		12		1		13	
Speech disorders		7		0		7	
Cosmetic problems		1		4		5	
Advised by parents or		4		0		4	
people around them							
Eating/suckling disorder		3		0		3	
Postresection recurrence		0		1		1	
Other reasons		0		2		2	
Total		27		8		35	

Frenectomy was performed using a CO_2_ laser in 21 patients (60%) with a mean age of 6 years (Table 3). Based on the site, 15 of 27 patients had lingual frenulum (55.6%; mean age, 5.2 years) and 6 of 8 patients had maxillary labial frenulum (75%; mean age, 8.2 years). Of the patients with lingual frenulum that underwent resection, 9 were type I, 6 were type II, and none were type III by Ito’s classifications. Of the patients with maxillary labial frenulum, 2 were type II, 4 were type III, none type I or VI by Rui’s classifications (Table 4). For 7 patients (mean age, 2.8 years), the procedure was performed under general anesthesia, whereas for 14 patients, the procedure was performed under local anesthesia (mean age, 7.6 years; Table 3). Suturing was performed in 7 of the 15 patients with lingual frenulum and in one of the 8 patients with maxillary labial frenulum. No problems intraoperatively occurred in any of the patients, and surgery was quickly and safely performed. No patients exhibited any adverse symptoms, such as hemorrhage or spontaneous pain after returning home. Readhesion occurred in one of the 15 patients with lingual frenulum (6.7% of resection patients) and in none of the patients with maxillary labial frenulum.

The follow-up observation period for the 21 patients who underwent resection ranged from 1 week to 3 years, and the mean follow-up period was 4.6 months (patients with lingual frenulum, 5 months; patients with maxillary labial frenulum, 1.6 months). The mean age of the 14 patients (39%) who did not undergo surgical treatment was 3.4 years (patients with lingual frenulum, 3.8 years; patients with maxillary labial frenulum, 1 year). Reasons for not undergoing resection were young age (6 patients; mean age, 1.2 year), no abnormal findings/ disability (5 patients; mean age, 5.5 years), and refusal of treatment under general anesthesia (3 patients; mean age, 4.3 years; Table 5).

**Table 3:** **Table 3:** Age distributions of lingual and maxillary frenulum patients

*Lingual frenulum*								*Maxillary frenulum*					
*Age*		*Total*		*Observation*		*Excision (under general anesthesia)*		*Total*		*Observation*		*Excision (under general anesthesia)*	
0		3		2		1 (1)		0					
1		1		1		0		2		2		0	
2		4		3		1 (1)		0					
3		5		1		4 (4)		0					
4		1		0		1 (0)		1		0		1(0)	
5		3		2		1 (1)		0					
6		2		1		1 (0)		0					
7		2		1		1 (0)		1		0		1(0)	
8		4		0		4 (0)		3		0		3(0)	
9~		2		1		1 (0)		1		0		1(0)	
Total		27		12		15 (7)		8		2		6(0)	

## DISCUSSION

### Patients with Frenulum Abnormalities Who were Examined at our Hospital

In this study, a clear difference in the number of patients with maxillary labial frenulum (n = 8) or lingual frenulum (n = 27) was observed. A common chief complaint for patients with maxillary labial frenulum was cosmetic problems.^8^ Because a tendency for reduction of maxillary labial frenulum is noted, with alveolar bone growth and tooth eruption, many general practitioners (GPs) choose to take a conservative approach for such cases. Meanwhile, lingual frenulum abnormalities tend to be considered more problematic because they can lead to functional disorders, such as speech or eating disorders.^9-11^ We believe that the reason for many such cases being referred to the Department of Oral and Maxillofacial Surgery (our hospital) as compared with those of labial frenulum is that many GPs are reluctant to treat sublingual soft tissue due to the technical difficulty.

### Timing and Reason for Consultation

Ankyloglossia caused by lingual frenulum abnormalities is mainly characterized by the fibrous adhesion of the tongue to the floor of the oral cavity. It is classified as complete or partial ankyloglossia depending on the extent of adhesion.^12,13^ Cases of complete ankyloglossia are rare, with most cases exhibiting partial ankyloglos-sia, in which the site of abnormal frenulum adhesion is either the body of the tongue or the mandibular alveolar mucosa.^12^ Very few cases in this study were classified as type III according to the Ito’s classifications (Table 4). Generally, ankyloglossia is detected at a young age due to dysphagia, masticatory difficulty, speech disorder, or at a regular checkup.^14,15^ In this study, 19 of the 27 patients (70.4%) were at our department by the time they reached school age (6 years or younger). The maxillary labial frenulum is connected to the incisive papilla during the early embryonic period. Subsequently, a gap gradually develops, and labial frenulum regresses with the development of the alveolar bone after birth and the eruption of deciduous incisors. At around 10 years of age, after the mixed dentition period (ugly duckling stage) has finished and the six maxillary anterior region teeth have erupted, the diastema naturally closes and stabilizes. Accordingly, it has been reported that as long as no clear functional disorder is noted, it is best to continue with regular follow-up examinations of the maxillary labial frenulum until around the age of 10 years.^16^ According to other reports in the literature, the most common reasons for seeking consultation are being advised to do so at regular checkups by dentist,^13^ followed by being referred by a doctor, coming to the hospital directly, being referred by a language workshop. In the present study, the most common reason was being advised to seek an examination at a regular checkup (Table 2). Regular checkups appear to be useful for early detection and treatment.

**Table 4:** **Table 4:** Age distribution analyzed by Ito’s and Rui’s classifications

		*Lingual frenulum*						*Maxillary frenulum*							
		*Ito’s classifications*						*Rui’s classifications*							
*Age*		*I*		*II*		*III*		*I*		*II*		*III*		*IV*	
0				1											
2		1													
3		1		3											
4		1										1			
5				1											
6		1													
7				1						1					
8		4								1		2			
9~		1										1			
Total		9		6		0		0		2		4		0	

**Table 5:** **Table 5:** Age range of reasons for not undergoing resection

*Reasons of observation*		*Number*		*Age range*		*Average age*	
Young age		6		lm~2y		1.2	
No abnormal findings		5		4m~12y		5.5	
Refusal of treatment		3		2~6y		4.3	
		14		lm~12y		3.4	

### Discussion of the usefulness of Laser Treatment

The energy emitted by a CO_2_ laser at the wavelength of 10.6 μm is efficiently absorbed into tissues with high moisture content, and this laser is commonly used for the resection and vaporization of soft tissues in the oral cavity.^3,12,17^ While many facilities tend to use a cold scalpel or an electric scalpel to perform frenectomy, a laser is utilized at our hospital.^18^ When using a scalpel, sutures are required for intraoperative hemorrhage, whereas the electric scalpel offers strong hemostatic effects by means of thick coagulation and deformation layers. However, the surrounding areas are affected by heat, and problems, such as enlarged wound, infection, delayed healing, and postoperative pain, are likely to occur, with many cases also requiring sutures.^18^ Meanwhile, although the CO_2 _laser has a shallow resection surface depth, it causes relatively no wound surface opening due to heat effects being localized, and coagulation/deformation layers being of appropriate thickness, thus implying reliable hemostasis and early healing.^5,19^ In an investigation using the visual analog scale, it was reported that postoperative pain and discomfort during mastication and speech were statistically and significantly less common with the CO_2_ laser than with a conventional scalpel.^18^ Thus, when compared with surgery using a cold or electric scalpel, the use of a laser can reduce treatment time and simplify the overall surgical procedure, reducing the burden on patients and making it easier to gain their cooperation. Therefore, it appears to be a highly useful in surgical procedures, such as frenectomy that are commonly performed on pediatric patients.^12,16^ However, when using this laser to treat maxillary labial frenulum abnormalities, attention must be paid to the power during laser application. In contrast to lingual frenectomy cases, excessive power can lead to damage of the bone surface, and constant care must be taken to protect the eyes because the laser is applied toward the upper lip.

It has been reported that the erbium-doped yttrium aluminum garnet (Er:YAG) laser, which, like the CO_2 _laser, is commonly used for oral soft tissue diseases, can be used without local anesthesia by performing irrigation of the application site, making it possible to perform procedures, such as frenectomy with surface anesthesia alone.^20^ However, as the Er:YAG laser has weaker hemo-static effects directly after resection than the CO_2_ laser, many cases require the use of local anesthetics containing vasoconstrictors.^21^

### Discussion of Treatment Standards

When the frenulum abnormality is causing difficulty in suckling, some reports recommend early resection of the lingual frenulum.^22,23^ However, numerous reports suggest avoiding performing resection on patients of a very young age due to the possibility of improvement with growth and the fact that the procedure can cause scarring.^24,25^ Furthermore, Tachimura et al^26^ suggested that it is important to first conduct an articulation training before determining resection timing based on the degree of development in articulation function. Thus, no fixed consensus has been reached regarding the optimal timing for resection at the present time. In this study, 15 patients that underwent lingual frenectomy (mean age, 5.2 years) and 12 patients that underwent conservative treatment (mean age, 3.8 years) were retrospectively investigated. Excluding the patients whose speech had not fully developed and the patients in whom postoperative tongue motion functional training was difficult, a trend toward performing lingual frenectomy from the age of 3 years and onward was noted. All resection cases in this study were classified as I to II (moderate or lower level of impairment of tongue range of motion or deformity) according to Ito’s classifications (Table 4), and surgery was selected for young patients aged 2 years or younger with suckling disorders in infancy or functional disorders in the growth process, such as marked speech disorders. Therefore, rather than relying upon systematic classifications, we determined whether or not to perform surgery at our hospital based on functional disorders as described by parents, who observed these problems on a daily basis. General anesthesia was selected for most procedures performed on patients aged 3 years or younger. This allowed the treatment to be smoothly performed and reduced the psychological stress and trauma of the patient. The disorders caused by maxillary labial frenulum abnormalities include diastema and abnormal central incisor position, onset of dental caries and periodontal disease due to the retention of food residue, and movement and cosmetic impairment of the upper lip. However, if no functional disorders, such as the above mentioned are clearly noted, it is best to take a conservative approach with regular follow-up observation. In the present study, the mean age of the six maxillary labial frenulum cases that underwent resection was 8 years, and 50% of these cases underwent the procedure as a preliminary treatment for subsequent orthodontic treatment. Unlike the lingual frenulum cases, none of the maxillary labial frenulum cases underwent resection with general anesthesia.

## CONCLUSION

Clinical data from 36 patients with frenulum abnormalities that were examined at the Department of Oral and Maxillofacial Surgery demonstrated the usefulness of the CO_2_ laser in performing frenectomy and offered suggestions regarding the timing of this surgical procedure.

## CLINICAL SIGNIFICANCE

Frenectomy performed using the CO_2_ laser in pediatric patients is a useful, simple, and safe treatment method that leads to good postoperative outcomes.
